# Genetic and protein interaction studies between the ciliary dyslexia candidate genes *DYX1C1* and *DCDC2*

**DOI:** 10.1186/s12860-023-00483-4

**Published:** 2023-05-26

**Authors:** Andrea Bieder, Gayathri Chandrasekar, Arpit Wason, Steffen Erkelenz, Jay Gopalakrishnan, Juha Kere, Isabel Tapia-Páez

**Affiliations:** 1 Department of Biosciences and Nutrition, Karolinska Institutet, Huddinge, Sweden; 2 Center for Molecular Medicine, Institute for Biochemistry I of the University of Cologne, Cologne, Germany; 3 Institute of Human Genetics, Universitätsklinikum, Heinrich Heine University, Düsseldorf, Germany; 4 Molecular Neurology Research Program, University of Helsinki, Folkhälsan Institute of Genetics, Helsinki, Finland; 5 Department of Medicine, Solna, Karolinska Institutet, Solnavägen 30, SE-171 76 Solna, Sweden

**Keywords:** Cilia, Centrosome, Dyslexia, Genetic interaction, Zebrafish

## Abstract

**Background:**

*DYX1C1 (DNAAF4)* and *DCDC2* are two of the most replicated dyslexia candidate genes in genetic studies. They both have demonstrated roles in neuronal migration, in cilia growth and function and they both are cytoskeletal interactors. In addition, they both have been characterized as ciliopathy genes. However, their exact molecular functions are still incompletely described. Based on these known roles, we asked whether *DYX1C1* and *DCDC2* interact on the genetic and the protein level.

**Results:**

Here, we report the physical protein-protein interaction of DYX1C1 and DCDC2 as well as their respective interactions with the centrosomal protein CPAP (CENPJ) on exogenous and endogenous levels in different cell models including brain organoids. In addition, we show a synergistic genetic interaction between *dyx1c1* and *dcdc2b* in zebrafish exacerbating the ciliary phenotype. Finally, we show a mutual effect on transcriptional regulation among *DYX1C1* and *DCDC2* in a cellular model.

**Conclusions:**

In summary, we describe the physical and functional interaction between the two genes *DYX1C1* and *DCDC2*. These results contribute to the growing understanding of the molecular roles of *DYX1C1* and *DCDC2* and set the stage for future functional studies.

**Supplementary Information:**

The online version contains supplementary material available at 10.1186/s12860-023-00483-4.

## Background

Developmental dyslexia (DD) is the most common neurodevelopmental learning disability. Its heredity has been established and the underlying genetic causes are beginning to emerge with the identification of dyslexia susceptibility genes [1]. Two of the most replicated dyslexia candidate genes are *DYX1C1 (DNAAF4)* and *DCDC2* [2–4]. *Dyx1c1* and *Dcdc2* have been shown to have roles in neuronal migration in rodent knockdown models and in *in vitro* models, reminiscent of early studies showing neuronal migration abnormalities in postmortem dyslexic brains [3, 5–8].

In addition to their shared association with DD, both *DYX1C1* and *DCDC2* have established roles in cilia and in ciliopathies. Cilia are microtubule-based organelles protruding from the cell surface of most eukaryotic cells and are anchored to the cell via the basal body, which grows from the mother centriole of the centrosome. Ciliopathies are a class of genetic disorders linked to malfunctioning of the cilium and in some cases involving neurodevelopmental phenotypes [9, 10]. Many ciliopathies display a brain phenotype, and a growing body of evidence is emerging for a role of cilia in neuropsychiatric disorders such as schizophrenia and autism [11–15]. Mutations in *DYX1C1* cause primary ciliary dyskinesia (PCD) and loss-of function mutations in *DCDC2* can cause a wide spectrum of cilia-related disorders such as nephronophthisis-related ciliopathies (NPHP-RC), hereditary hearing loss, and neonatal sclerosing cholangitis [16–21]. Several high-throughput transcriptomics studies show that *DYX1C1* and *DCDC2* are upregulated in highly ciliated tissues [22–24]. Furthermore, knockdown of *dyx1c1* in zebrafish embryos produces a broad ciliary phenotype and *dyx1c1* mutant zebrafish display idiopathic scoliosis due to motile ciliary defects [16, 25, 26]. Similarly, zebrafish morphants of *dcdc2b* display a ciliopathy phenotype including curved body, pronephric cysts and left-right asymmetry defects [18]. DYX1C1 has been proposed as a cytoplasmic axonemal dynein assembly factor [16, 27–30]. Recent studies have strengthened a role of *DCDC2* and *DYX1C1* also in primary cilia and human neurons [31, 32].

We have shown previously by proteomic studies that DYX1C1 interacts with CPAP (Centrosomal-P4.1-associated protein, Centromere protein J (CENPJ)) in the neuroblastoma cell line SH-SY5Y [7]. *CPAP* was identified as a regulator of brain size and its mutation causes autosomal recessive primary microcephaly (MCPH) and Seckel syndrome [33, 34]. It is required for cilia formation in neuronal cells *in vitro* and is crucial for cilia length control, cilia disassembly, cell cycle reentry, neural stem cell maintenance and microtubule regulation [35–40]. In addition to this well-studied centrosomal role, a role in neuronal migration has been described, downstream of the neurogenic transcription factor *Ascl1/Mash1* [41]. Indeed, knockdown of *Cpap* in mouse produces a neuronal migration phenotype reminiscent of *Dyx1c1* and *Dcdc2* knockdown phenotypes in rats [3, 6, 41].

It is important to understand the relationship among dyslexia candidate genes and their interacting partners and their relation to ciliary processes. Based on the results that, 1) *DYX1C1* and *DCDC2* are both dyslexia candidate genes, 2) they are both involved in neuronal migration and 3) they are both involved in ciliary processes, we asked whether there is a functional relationship between *DYX1C1* and *DCDC2*. Here, we study a possible functional interaction between DYX1C1 and DCDC2 by using protein-protein interaction, subcellular localization and perturbation approaches *in vitro* and a genetic interaction model *in vivo*. We show on exogenous and endogenous levels that the CPAP protein interacts with both DCDC2 and DYX1C1 in human cell models, supporting further an interacting role of DYX1C1 and DCDC2. Building on the previous results with loss-of-function experiments in zebrafish [16, 18, 25], we show that a combined knockdown of *dcdc2b* and *dyx1c1* produces an exacerbated phenotype suggesting a synergistic effect between these two genes. In addition, we show mutual transcriptional regulation of *DYX1C1* and *DCDC2* in the hTERT-RPE1 cell line.

In summary, we demonstrate an interaction between DYX1C1, DCDC2 and CPAP on the gene and protein levels.

## Results

### CPAP interacts with both DCDC2 and DYX1C1 proteins

**Fig. 1:**
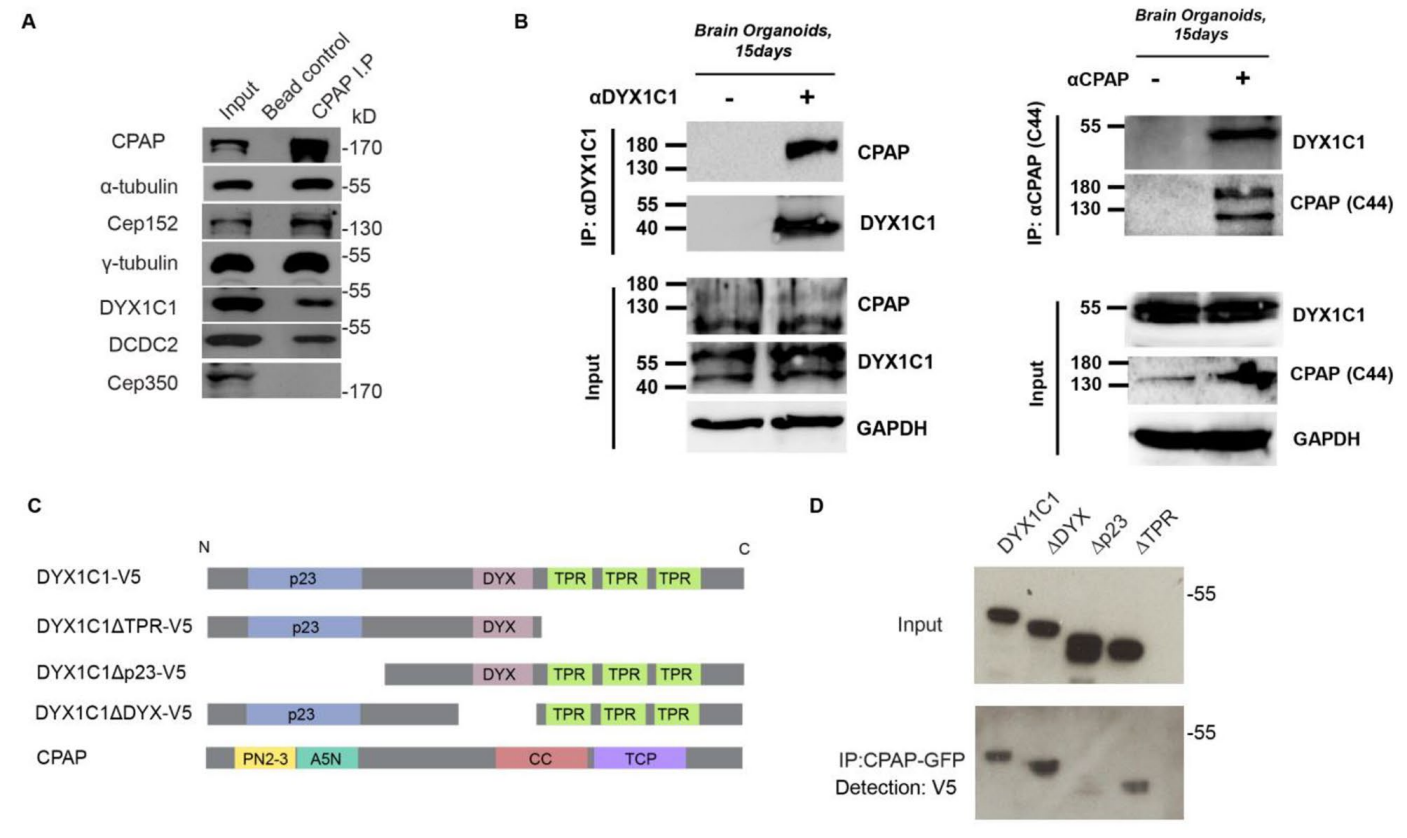
CPAP interacts with DCDC2 and DYX1C1. (**A**) CPAP endogenously interacts with DCDC2 and DYX1C1. Endogenous immunoprecipitations using HeLa cell extracts. CPAP was used as a bait to pull down interactors. CEP350 was used as a negative control and CEP152 was used as a positive control for interaction with CPAP. Beads alone were used as a negative control for the IP´s. (**B**) CPAP and DYX1C1 interaction in brain organoids. CPAP was immunoprecipitated by anti- DYX1C1 in 15 day old brain organoids. Reciprocally, DYX1C1 was pulled down by anti-CPAP. (**C**) Schematic representation of domain structures of DYX1C1, CPAP and the deletion constructs DYX1C1ΔTPR, DYX1C1Δp23, DYX1C1ΔDYX. p23 = p23 domain, TPR = tetratricopeptide repeat domain, DYX = DYX domain. (**D**) CPAP interacts with DYX1C1 via the p23 domain. hTERT-RPE1 cells stably expressing DOX-CPAP-GFP and growing in normal serum conditions were induced with doxycycline and transiently transfected with the indicated constructs. GFP-Trap was used to pull down CPAP-GFP. anti-V5 antibody was used for immunodetection of interactors.

We have previously observed a protein-protein interaction between DCDC2 and DYX1C1 by pulldown approaches in SH-SY5Y cells. In addition, we observed an interaction between CPAP and DYX1C1 by LC/MS-MS in SH-SY5Y cells [7]. Here, we sought to confirm the interaction between DYX1C1 and CPAP by co-immunoprecipitation and asked whether DCDC2 also interacts with CPAP. First, we investigated whether these protein-protein interactions can be observed endogenously. We performed immunoprecipitations using HeLa whole cell extracts. α-tubulin, γ-tubulin and CEP152 were included as positive controls for interaction with CPAP and CEP350 as a negative control for interaction with CPAP [35]. Indeed, we observed a protein-protein interaction between DYX1C1 and CPAP and between DCDC2 and CPAP on endogenous protein levels (Fig. 1A). As both DYX1C1 and CPAP have roles in brain development, we next tested if there is an interaction in 15 day old hiPSC-derived brain organoids. Indeed, we observed successful immunoprecipitation of CPAP using a DYX1C1 antibody and, reciprocally, pulldown of DYX1C1 using a CPAP antibody (Fig. 1B).

Next, we asked which protein domains in DYX1C1 are mediating these interactions. We used the stable doxycycline-inducible cell line hTERT-RPE1-DOX-CPAP-GFP, where CPAP is epitope-tagged to GFP. We transfected doxycycline-induced cells growing in normal serum conditions with the epitope-tagged expression constructs DYX1C1-V5 and DCDC2-V5 and used whole cell extracts to perform immunoprecipitations. We found that both DYX1C1-V5 and DCDC2-V5 were successfully pulled down by the GFP-trap (Fig. 1D). The p23 and TPR (tetratricopeptide repeat) domains have previously been reported to be crucial in protein-protein interactions, while the DYX domain was identified as a novel, highly conserved domain specific for DYX1C1 [7, 42–45]. We explored which of the domains in DYX1C1 mediates the interaction with CPAP by using epitope-tagged expression constructs with deletions in defined domains for transfection: DYX1C1ΔDYX-V5, DYX1C1ΔTPR-V5, DYX1C1Δp23-V5 (Fig. 1C). We found that DYX1C1 interaction with CPAP was abrogated upon deletion of the p23 domain, but not the TPR or the DYX domains. We concluded that DYX1C1 interacts with CPAP via the p23 domain (Fig. 1D).

In summary, we confirm the previously reported protein-protein interaction between DYX1C1 and CPAP, characterize their interacting domain and report a novel interaction between DCDC2 and CPAP.

### Co-occurrence of DYX1C1 and CPAP at the centrosome

**Fig. 2:**
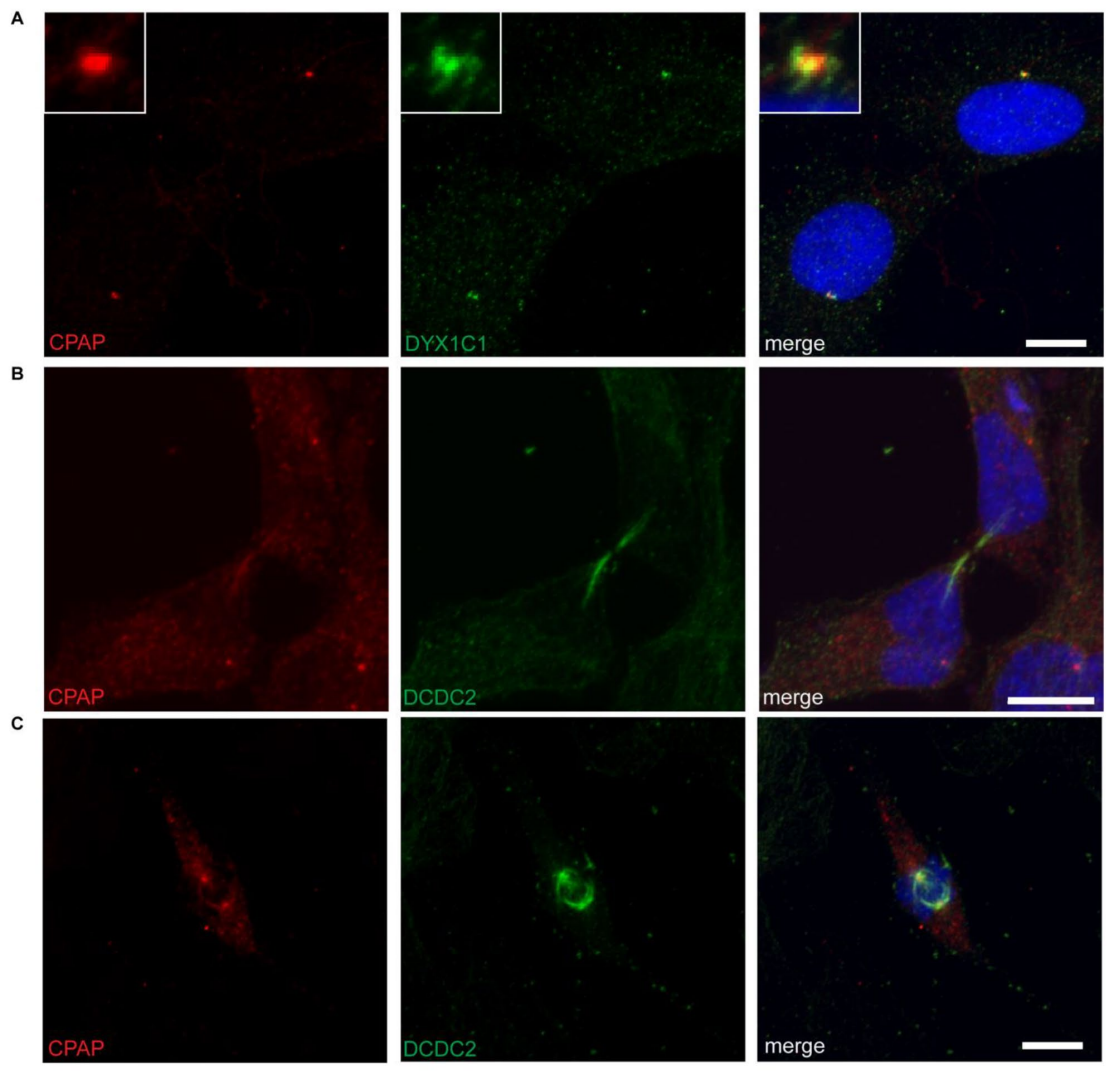
Co-occurrence of DYX1C1 and CPAP at the subcellular level. **A**) DYX1C1 and CPAP co-occur around the centrosome. Confocal immunofluorescence images of endogenous protein in hTERT-RPE1 cells grown in normal serum conditions. **B**)-**C**) DCDC2 co-occurs with CPAP depending on cell cycle stage. Nuclei are stained with DRAQ5. Scale bar = 10 μm

Several studies have reported a localization of DYX1C1 at or around the centrosome [6, 7, 24, 31, 46]. We therefore hypothesized that DYX1C1 and CPAP might co-occur at the centrosome. We performed immunofluorescence on hTERT-RPE1 cells grown in normal serum conditions. Indeed, we observed a co-occurrence of DYX1C1 and CPAP at the centrosome (Fig. 2A). DCDC2 has been reported to dynamically localize to the ciliary axoneme, the abscission structure (midbody) and the mitotic spindle fibers [18]. DCDC2 does not co-occur with CPAP during cytokinesis (Fig. 2B). During metaphase, DCDC2 is at the centrosomes, where it co-occurs with CPAP, and at the mitotic spindle (Fig. 2C).

In summary, we observed that DYX1C1 co-occurs with CPAP at the centrosome, while DCDC2 localization depends on cell cycle stages and may co-occur with CPAP during certain stages.

### Exacerbated ciliary phenotype by double morpholino knockdown of *dyx1c1* and *dcdc2b* in zebrafish

**Fig. 3:**
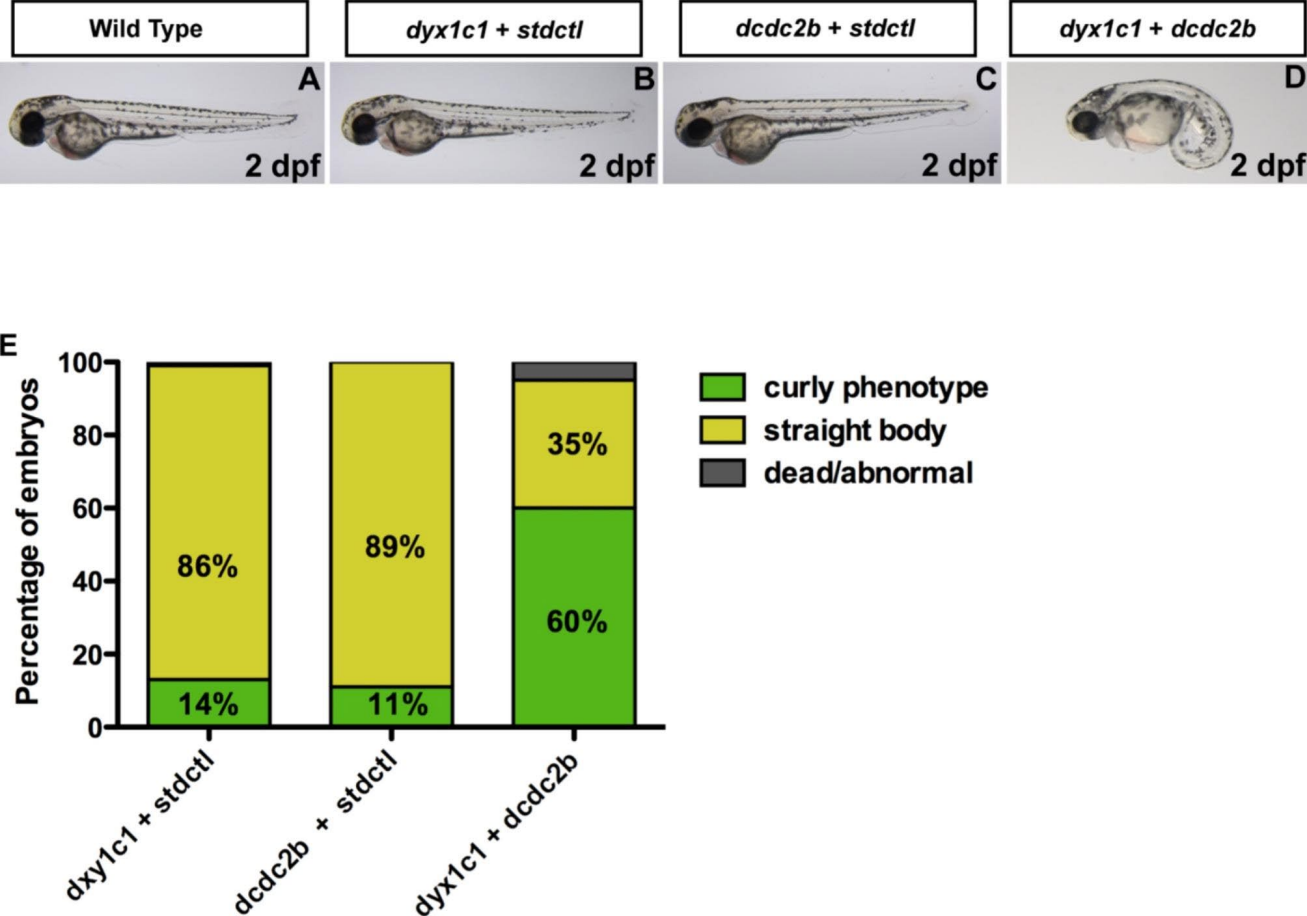
Genetic interaction between *dyx1c1* and *dcdc2b* in zebrafish. A,B,C) Injection of 20 µM of *dyx1c1*MO or 100 µM of *dcdc2b*MO individually in zebrafish embryos resulted in a maximum number of embryos with straight body axis similar to wildtype embryos at 2dpf. D) Co-injection of the subphenotypic doses of *dyx1c1MO* and *dcdc2bMO* (20 µM *dyx1c1* + 100µM *dcdc2b*) resulted in a strong curved body axis in a greater number of embryos at 2dpf. StdctlMO was used in order to maintain a uniform concentration of the morpholinos. E) Graphical representation of percentage of embryos with strong curly phenotype following MO injection. Coinjection of *dyx1c1* and *dcdc2b* resulted in a dramatic increase in number of embryos with strong curly body axis as compared to individual knockdown of the two genes. The number of embryos examined in control and knockdown groups were 100 (n = 100).

Our previous *in vitro* analyses support the interaction between the two proteins DYX1C1 and DCDC2 [7], (this study). In addition, strong ciliary phenotypes including ventrally curved body axis, hydrocephalus, *situs inversus* and kidney cysts have been observed in zebrafish following morpholinobased knockdown of *dyx1c1* or *dcdc2b* [18, 25]. Moreover, a growing body of genetic and functional evidence suggests digenic interactions among ciliopathy genes, which can be addressed by using combined morpholino knockdowns in the zebrafish [47–51]. To investigate whether there is a digenic interaction between *dyx1c1* and *dcdc2b in vivo*, we performed morpholino coinjection studies in zebrafish embryos and examined their phenotypes. A subphenotypic dose of 20 µM for *dyx1c1* and 100 µM for *dcdc2b* was chosen for coinjection experiments as they did not produce any ciliary phenotype when injected either alone or in combination with 100 µM or 20 µM of stdctlMO, respectively, into 1-cell stage embryos. Interestingly, when a combined dose of 20 µM of *dyx1c1* and 100 µM of *dcdc2b* was injected, a dramatic exacerbation of the phenotype was observed in 60% of the embryos injected with the morpholinos (Fig. 3). These results suggest a genetic interaction between the two genes *in vivo.* In summary, we observed that the two genes *dyx1c1* and *dcdc2b* synergize to produce an exacerbated ciliary phenotype in zebrafish.

### Transcriptional regulation between *dyx1c1/DYX1C1* and *dcdc2* / *DCDC2*

**Fig. 4:**
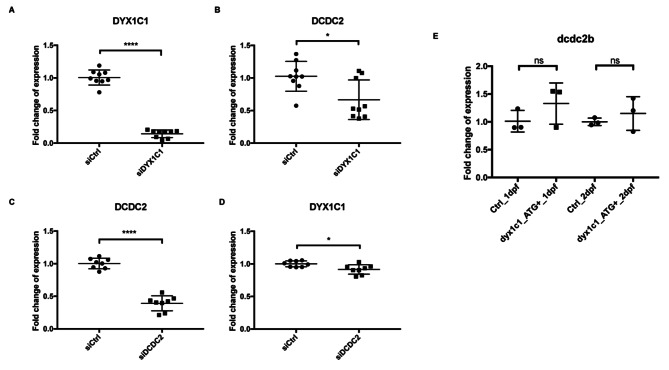
Transcriptional control between *DYX1C1/dyx1c1* and *DCDC2/dcdc2b.***A**-**D**). hTERT-RPE1 cells were transfected with siDYX1C1, siDCDC2 or siCtrl, respectively, RNA was extracted and qRT-PCR was run (n = 3 experiments with 3 replicates, mean ± sem). **A**, **C**) Knockdown of *DYX1C1* and *DCDC2* efficiently reduced the respective transcripts (p-value < 0.0001, t-test). **B**, **D**) Expression of *DCDC2* and *DYX1C1* is reduced after knockdown of siDYX1C1 and siDCDC2, respectively (p-value < 0.05, t-test). **E**) *dcdc2b* expression was unchanged in *dyx1c1* morphants: RNA of *dyx1c1* morphant zebrafish was collected at 1dpf and 2dpf after morpholino injection at 1-cell stage. RT-qPCR analysis shows that *dcdc2b* expression is unchanged in *dyx1c1* morphant embryos compared to control embryos (n = 3, mean ± sem, p-value (1dpf) = 0.2597, p-value (2dpf) = 0.4521), t-test). dpf = days post fertilization

Next, we asked whether there is transcriptional regulation between *dyx1c1/DYX1C1* and *dcdc2b*/*DCDC2*. We knocked down *DYX1C1* and *DCDC2* in the human ciliated hTERT-RPE1 cell line using siRNA, which efficiently reduced expression of *DYX1C1* and *DCDC2* (Fig. 4A, C). We observed that *DCDC2* expression was significantly downregulated upon knockdown of *DYX1C1* (Fig. 4B). However, overexpression of *DYX1C1* did not alter *DCDC2* expression in hTERT-RPE1 cells (data not shown). Similarly, knockdown of *DCDC2* led to a downregulation of *DYX1C1* (Fig. 4D).

We also collected RNA from zebrafish at 1 dpf and 2 dpf after *dyx1c1 -*morpholino injection into 1-cell stage embryos and performed qRT-PCR analysis to measure the expression of *dcdc2b*. *dcdc2b* expression was not altered in *dyx1c1* morphant embryos compared to control embryos at 1 dpf and at 2 dpf (Fig. 4E). In summary, we observed that *dcdc2b* expression is unchanged in the *dyx1c1* morpholino injected zebrafish, but that expression of *DCDC2* and *DYX1C1* are changed upon knockdown of *DYX1C1* and *DCDC2*, respectively, in a human ciliated cell line. Taken together, these results suggest that there is transcriptional control among *DYX1C1* and *DCDC2* in certain cell types indicating that *DYX1C1* and *DCDC2* may act in common transcriptional pathways in a tissue-specific manner.

## Discussion

In recent years, the detailed molecular functions of *DYX1C1* and *DCDC2* have been emerging. Both genes have been assigned roles in developmental dyslexia, neuronal migration and cilia. Given these overlapping functions, the question arises whether there is a functional relationship between these two genes.

Here, we show that DYX1C1 and DCDC2 proteins both interact with CPAP. We validate the previously suggested protein-protein interaction between CPAP and DYX1C1 by co-immunoprecipitation and identify the p23 domain as the interaction-mediating domain. This result is consistent with a previous study showing protein-protein interactions of DYX1C1 mainly via p23 [7]. P23 domains are known to be important for protein-protein interactions, for example with the chaperone Hsp90 [43–45]. In addition, it is known that DYX1C1 is interacting with Hsp70 and Hsp90 and has a chaperone function [16, 52, 53]. TPR domain containing proteins (TTC proteins) have been reported to have important roles in cilia functioning in IFT and dynein assembly [54]. A ciliary function of the CCT/TRiC chaperone complex has previously been described affecting the BBSome [55]. Interestingly, it has been shown that Dyx1c1 interacts with the chaperones Cct3, Cct4, Cct5 and Cct8 while CCT4, CCT5 and CCT8 are reportedly localizing to the centrosome [16, 55]. It remains to be determined whether the chaperone-functions and cilia-related functions of DYX1C1 are disparate or functionally related.

Both *Dyx1c1* and *Dcdc2* have been shown to produce neuronal migration defects when knocked down in rats [3, 6]. Similarly, CPAP has been shown to regulate neuronal migration independently of its function at the centrosome, via its microtubule-destabilizing domain PN2-3 [41]. CPAP controls, via its PN2-3 domain, ciliary length and centriolar and ciliary tubulin assembly and disassembly [39, 56–58]. CPAP promotes cilia disassembly thereby keeping a neural stem cell pool [35]. *Ascl1*— a proneural transcription factor known for its role in neurogenesis — is a transcriptional regulator of *Cpap*, while *DYX1C1* perturbation affects *ASCL1* expression [7, 41, 59]. This indicates a complex regulatory mechanism of *CPAP* by *DYX1C1* and *ASCL1*. *FOXP2*, a gene often associated with speech and language disorders, affects *DCDC2* expression in SH-SY5Y cells providing a further link between language-related gene expression and *DCDC2* [60]. Interestingly, *DYX1C1* is highly upregulated in differentiating human neurons [32]. Further studies in human stem cell derived neurons might shed more light on the role of these genes in neuronal migration.

Both DYX1C1 and DCDC2 have been reported as cytoskeletal interactors [7, 61]. DYX1C1 associates with microtubule proteins and DCDC2 has a role in microtubule stabilization [7, 61]. DCDC2 localizes to primary cilia and is involved in ciliary signaling [18, 62]. Some studies report a physical localization of DYX1C1 at or around the basal body [6, 7, 31, 46]. It might be a dynamic process, shuttling between the cytoplasm and temporal accumulation around the basal body. DCDC2 and DYX1C1 do not co-occur at the cilium and our immunoprecipitations were carried out on non-starved, cycling cells both suggesting that their combined action may take place in the cytoplasm, consistent with in situ proximity ligation assay (PLA) data [7]. Future studies using PLA might pinpoint the subcellular localization of their interaction with CPAP.

Genetic interaction studies have been successfully used to study cellular pathways in model systems, for example zebrafish [63]. They can provide useful information about protein-protein complexes, downstream pathways and parallel pathways. This approach has been used to dissect interactions of ciliary genes – for example, the genetic interactions of BBS and PCD genes have been modelled in zebrafish [47–50]. Exacerbation of a ciliary phenotype can take place among physically interacting and among non-interacting proteins. For example, concomitant loss of bbs7 and bbs1 – members of the BBsome protein complex – and of dnah6 with dnai1 and dnah5 - where no physical interaction takes place – produces an aggravated phenotype [48, 49]. Previous studies have reported a ciliary phenotype in *dyx1c1* and *dcdc2* morphant zebrafish [18, 25]. Here, we show an exacerbated phenotype in double *dyx1c1* and *dcdc2b* morpholino injected zebrafish. The tissue expression specificity as well as the subcellular localization might overlap only partially raising the question whether dyx1c1 and dcdc2 might act in parallel pathways thereby aggravating the ciliary phenotype. Future studies should address whether *dyx1c1* morphants can be rescued by *dcdc2b* overexpression and vice-versa.

We show a mutual transcriptional regulation between *DYX1C1* and *DCDC2* in the human ciliated RPE1 cell line, but not in the morphant zebrafish. This might be due to dilution of the effect in the whole organism as compared to an isolated cell type. Transcriptional control likely is indirect as there is no evidence that either proteins would act as transcription factors. The regulation of *DCDC2* by *DYX1C1* has not been reported previously [7, 62, 64]. This might point to a cell-specific or cilia-specific effect, which could be further studied using starved and non-starved cells as well as different non-ciliated and ciliated cell lines.

Our study stands out in applying many different model systems, from different cell lines to brain organoids to zebrafish. We are the first to address a possible functional link between *DYX1C1*, *DCDC2* and *CPAP* by combining different experimental readouts. However, some limitations need to be considered: the DCDC2-CPAP interaction needs further dissection to determine the interacting domain of DCDC2. In the zebrafish, a rescue experiment would be needed in order to strengthen the case. In the cell models, cellular localization upon perturbation should be studied. Transcriptional regulation of *DYX1C1* and *DCDC2* will have to be confirmed by rescue experiments via overexpression of knockdown-resistant *DYX1C1*. These are some experiments that may help to further strengthen the notion that DYX1C1, DCDC2 and CPAP act via a common pathway.

## Conclusions

In conclusion, we report genetic- and protein interactions among DYX1C1, DCDC2 and CPAP. We thereby contribute to the growing understanding of the molecular roles of *DYX1C1* and *DCDC2*. Recently, studies combining patient cohorts, cellular models, zebrafish and mouse behavior assays have elegantly demonstrated the link between ciliary and centrosomal defects and neuropsychiatric disorders such as schizophrenia [14, 15]. Future studies should make use of a combination of genetic, behavioral and molecular approaches in order to provide a more integrative picture of the roles of ciliary dyslexia candidate genes in homeostasis and disease.

## Methods

### Zebrafish maintenance

Wild-type zebrafish were obtained from the zebrafish core facility at Karolinska Institutet and were reared and maintained as described previously [65]. Briefly, zebrafish were maintained at a temperature of 28^o^C and were kept under a 14:10 h light:dark cycle. They were fed daily with fish flakes and live brine shrimp. For breeding, one female and one male AB strain fish were placed in a breeding tank and were separated by a divider. When the lights turned on the following morning, the divider was removed to allow the fish to breed. Embryos that were obtained were collected and transferred to a petri dish containing E3 medium (5 mM NaCl, 0.17 mM KCl, 0.33 mM CaCl_2_, and 0.33 mM MgSO_4_). Methylene blue (0.1%) was added to the medium to prevent fungal contamination. Embryos were maintained at 28 °C until they reached the desired developmental stage. Developmental staging is shown as days post fertilization (dpf).

### Zebrafish embryos and microinjections of morpholinos and mRNA

Morpholinos against zebrafish *dyx1c1* and *dcdc2b* were designed as described previously [18, 25]. The following morpholinos (MO) were purchased from Gene Tools, LLC. *dyx1c1* AUGMO: 5’-GTGATCTCTCACTATCAGCGGCATC-3’, *dyx1c1* spliceMO: 5’-TGACAGTCAACATGTCTTACCGATG-3’, *dcdc2b* AUGMO: 5’-CCGGTGGATGCCATGACTTTTCAGT-3’ and standard control morpholino (stdctlMO): 5’-CCTCTTACCTCAGTTACAATTTATA-3’. Individual knockdown of *dyx1c1* and *dcdc2b* genes have been reported previously [18, 25]. Wildtype AB embryos of 1-cell stage were placed in furrows on a 2% agarose plate and a subphenotypic dose of 20 µM of *dyx1c1* together with 100 µM of *dcdc2b* was injected into them to study the synergistic effect of the two genes. As controls, a combined dose of 20 µM of *dyx1c1* plus 100 µM of stdctl or 100 µM of *dcdc2b* plus 20 µM of stdctl was injected into 1-cell stage embryos in order to keep the final concentration at 120 µM. After injection, the embryos were transferred to petri dishes with E3 medium and raised in an incubator maintained at 28^o^C until they were ready to be imaged. Dead and uninjected embryos were discarded and E3 medium was changed daily. Two days post fertilized embryos were examined for phenotype scoring and later anesthetized with tricaine (MS-222, 40 µg/ml) for imaging. Wild-type embryos were imaged and used as uninjected controls. After imaging all embryos were euthanized using an overdose of MS-222.

### Cell culture

The human retinal pigmented epithelial cell line immortalized with hTERT (hTERT-RPE1, ATCC, CRL-4000™) was cultured in DMEM/F12, 10% fetal bovine serum (FBS), 100 U/ml penicillin and 100 µg/ml streptomycin, 0.01 mg/ml hygromycin B at 5% CO_2_. To induce ciliogenesis, cells were starved using OptiMEM reduced serum medium for 24 h unless indicated otherwise. The doxycycline-inducible CPAP-GFP-hTERT-RPE1 cell line was described previously and cultured as described above [39]. CPAP expression was induced using 2 µg/ml of doxycycline for 2–12 h. CPAP-GFP expression was verified on an inverted fluorescence microscope (Olympus). Transient transfections were done using lipofectamine 2000 (Thermo Fisher Scientific). HeLa cells were cultured in a DMEM medium containing 10% FBS, 0.1 mM MEM non-essential amino acids (NAA), 100 µg/ml streptomycin, 100U/ml penicillin (Life Technologies GmbH, Darmstadt, Germany). hiPSC-derived brain organoids were cultured as described previously [35] and were differentiated for 15 days.

### Expression constructs

Cloning and validation of the expression constructs DYX1C1-V5, DCDC2-V5, DYX1C1ΔDYX-V5, DYX1C1ΔTPR-V5, DYX1C1Δp23-V5 in pcDNA3.1/V5-His-TOPO vector were described previously [7, 62, 66, 67].

### siRNA knockdown

hTERT-RPE1 cells were seeded in six-well plates and grown to 80% confluency. Cells were transfected at a final concentration of 25 nM of each siGENOME SMARTpool targeting DYX1C1 (Cat. Nr. M-015300-02) or DCDC2 (Cat. Nr. M-020868-01) and non-targeting control (Cat. Nr. D-001206-14-20) (all Thermo Scientific, Dharmacon) using Lipofectamine 2000 (Thermo Fisher Scientific). After 24 h, hTERT-RPE1 cells were serum-starved for 24 h and harvested. RNA was prepared using the NucleoSpin RNA kit (Macherey-Nagel) according to the manufacturer’s instructions. All the experiments were performed in triplicates and were repeated independently three times.

### Quantitative real-time PCR (qRT-PCR)

cDNA was synthesized with Maxima Kit (Thermo scientific) using 500 ng of RNA. qRT-PCR was analyzed with cDNA diluted 1:5 using Taqman expression assays and TaqMan fast Universal PCR Master Mix (Life Technologies, 4,352,042) (*DYX1C1*: Hs00370049_m1; *DCDC2*: Hs00393203_m1; *HPRT1*: Hs02800695_m1). *HPRT1* was used as an internal control to normalize expression levels. For zebrafish, SYBR Green primers were used (*dcdc2b*: F:ATG ACA CGT CAG CTC CAC AG; R: TGG AAT GGT GTG ACT CGC TC; *β-actin*, F-5′-TGC CCC TCG TGC TGT TTT-3′, R- 5′-TCT GTC CCA TGC CAA CCA T-3′) and FastStart Universal SYBR Green Master Mix (04913850001, Roche). The reactions were amplified using the 7500 Fast Real-Time PCR system (Applied Biosystems). The ΔΔCt method was used to calculate relative expression levels and shown as fold change (2^−∆∆Ct^)[68]. Student’s t-test was used for statistical analysis between conditions using GraphPad Prism 7 software.

### Immunoprecipitations

Figure 1 A: The endogenous immunoprecipitations were performed with the mouse monoclonal anti-CPAP antibody [39], coated beads or IgG control beads. Protein G beads were coated with anti-CPAP antibodies overnight at 4 °C, mixed with HeLa cell extracts, and incubated at 4 °C for 4 h. Cell extracts were prepared in a buffer containing 80 mM BRB, 100 mM KCl, 1 mM MgCl2, 1 mM EGTA, protease inhibitor cocktails, and 1 mM PMSF. The extracts were centrifuged at 100,000 g, and the supernatant was collected. These high-speed extracts were then used for immunopurification. The protein-bound beads were washed with BRB buffer containing 0.1% Triton X-100 and 100 mM NaCl and washed twice with highsalt buffer containing 500 mM salt. After a final wash with buffer containing 100 mM NaCl, the samples were eluted using Laemmli buffer for Western blot analyses. Figure 1B: Brain organoid samples were immediately lysed in 50 mM Tris–HCl (pH 7.8), 150 mM NaCl, 1 mM EDTA (pH 8.0), 1% Triton X-100, 0.01% Igepal and protease inhibitors (Roche Applied Science Cat #11 873 580 001). Subsequently, samples were cleared of debris and DNA by centrifugation (12 000 × g at 4 °C for 10 min). Protein concentrations were determined by Bradford assay (Roth GmbH Cat# K015.1) according to manufacturer’s instructions. For immunoprecipitation experiments, 2500 µg of total protein were incubated overnight at 4 °C with protein G Dynabeads (Invitrogen Cat# 10004D) coupled to CPAP (Hybridoma C44) or DYX1C1 (Proteintech, Cat# 14522-1-AP) antibody. After five washes in lysis buffer containing 150 mM NaCl, bound proteins were eluted with 0.1 M glycine–HCl (pH 3.0) for 5 min followed by neutralization with 0.5 M Tris–HCl (pH 7.8) and 1.5 M NaCl. Figure 1D: Immunoprecipitations were performed using GFP-trap. Cells were first lysed using 200 µl ice-cold Lysis buffer (10 mM Tris/Cl pH 7.5; 150 mM NaCl; 0.5 mM EDTA; 0.5% NP-40) containing protease inhibitors (Roche). Beads were equilibrated by adding 25 µl of bead slurry into 500 µl ice-cold buffer (10 mM Tris/Cl pH 7.5; 150 mM NaCl; 0.5 mM EDTA). Proteins were bound by adding the diluted lysate to the equilibrated GFP-trap beads, and washed 3 times with 500 µl of ice-cold dilution buffer. The experiments were repeated at least three times; a representative example is shown.

### SDS-PAGE and Western blotting

Figure 1 A: Protein samples were resolved in 8% or 12% acrylamide gels and transferred to nitrocellulose membranes. After incubating with primary antibodies overnight at 4 °C, the blots were treated with secondary antibodies at RT for 1 h. Super Signal West Pico or Femto Chemiluminescent substrates (Pierce) were used for detection. Antibodies for Western blots: monoclonal mouse anti-CPAP (1:50), mouse anti-γ-tubulin (1:3,000; Sigma-Aldrich), mouse anti-α-tubulin (1:3,000; Sigma), rabbit anti-Cep152 (E. Nigg), anti-Cep350, anti-DYX1C1, anti-DCDC2, and peroxidase-conjugated secondary antibodies (1:3,000; Life Technologies). Figure 1B: Samples were resolved by 10% SDS-PAGE. Target proteins were detected by immunoblotting with primary antibodies: mouse anti-CPAP (1:50, Hybridoma C44), rabbit anti-DYX1C1 (1:1.000, Proteintech Cat# 14522-1-AP) and mouse anti-GAPDH (1:10.000, Proteintech Cat# 60,004-I-Ig) followed by incubation with the corresponding goat-anti-mouse (1:5000, Thermo Fisher Scientific Cat#626,520) and donkey anti-rabbit (1:5000, Thermo Fisher Scientific Cat#A16023) HRP-coupled secondary antibodies. Figure 1D: Cells were lysed in NP40 cell lysis buffer (Thermo Fisher Scientific) containing protease inhibitors cocktail (Complete Mini, Roche, #11,836,153,001). The samples were then passed several times through a 26-gauge needle, placed on ice for 10 min and centrifuged in a pre-cooled microcentrifuge. Protein concentrations were determined using the Quant-iT Protein Assay Kit and Qubit fluorometer (Thermo Fisher Scientific). Gel electrophoresis was performed using a 4–12% gradient gel (Invitrogen Bis-Tris NuPAGE gels) and proteins were transferred to a Amersham Hybond-P nylon membrane (GE healthcare). The transfer was done with a semi-dry transfer apparatus (Biorad) at 20 V for 1 h using transfer buffer (25mM Tris, 190 mM glycine, 20% MeOH, 0,05% SDS). The blot was then incubated overnight at 4 °C with blocking buffer (5% non-fat dry milk, 10 mM Tris pH 7.5, 100 mM NaCl, 0.1% Tween 20 PBS). Probing was done using anti-V5-HRP antibody (Invitrogen, 1:8000) overnight at 4^o^C and a secondary anti-HRP antibody (Invitrogen, 1:10^6^). Detection was performed with Luminol Enhancer Solution and Peroxide Solution (Thermo Fisher Scientific) for 5 min.

### Immunofluorescence

hTERT-RPE1 cells were seeded on coverslips, grown to 80% confluency and fixed. (For ciliary stainings, cells additionally were serum-starved for 24 h using OptiMEM reduced serum medium before fixation). Cells were incubated on ice for 45 min, then fixed in a 50:50 solution of 4% formaldehyde (Thermo Fisher Scientific, Prod. Nr. 28,906) and methanol or in pure methanol for 15 min at -20 °C. The fixed cells were blocked and permeabilized in 5% horse serum (Life Technologies), 0.05% PBS-Tween for 1 h at room temperature and incubated o/n at 4 °C with a primary antibody (rabbit anti-DYX1C1, 1:500, Sigma, SAB4200128[16]; rabbit anti-DCDC2, 1:200, Abcam, ab157186[18]; mouse anti-CPAP (Gopalakrishnan lab), 1:20; mouse anti-acetylated tubulin, 1:5000, Sigma T7451; mouse anti-gamma-tubulin, 1:500, Abcam, ab11316). Antibodies have been previously validated for specificity [16, 18, 35]. Cells were incubated for 1 h at room temperature with secondary antibodies (donkey anti-rabbit 568, Invitrogen, A10042; donkey anti-mouse 568, Invitrogen, A10037). Nuclei were stained with DRAQ5 (Cell Signaling Technology, Cambridge, UK) at 1:1000 for 10 min at room temperature and coverslips were mounted in Prolong Gold antifade reagent (Invitrogen). Images were acquired on a A1R confocal (Nikon Instruments, Inc.) using NIS elements software. Brightness and contrast were adjusted using the NIS elements software and the far-red channel was pseudo colored in blue.

## Electronic supplementary material

Below is the link to the electronic supplementary material.

Supplementary Material 1

## Data Availability

Not applicable.

## List of abbreviations

DCDC2: Doublecortin domain containing 2 gene
DD: Developmental dyslexia
DYX1C1/DNAAF4: Dyslexia 1 candidate 1 gene/ Dynein axonemal assembly factor 4
hTERT-RPE1: human retinal pigmented epithelial cell line immortalized with hTERT
CENPJ/CPAP: Centrosomal protein J/ Centrosomal-P4.1-associated protein
PCD: primary ciliary dyskinesia
PLA: in situ proximity ligation assay
NPHP-RC: nephronophthisis-related ciliopathies
BBS: Bardet-Biedl-Syndrome
TPR: tetratricopeptide repeat domain
