# Supplementary material for: Genetic and protein interaction studies between the ciliary dyslexia candidate genes DYX1C1 and DCDC2
Source: BMC Mol Cell Biol. 2023 May 26;24:20. doi: 10.1186/s12860-023-00483-4 (PMC10224228; doi:10.1186/s12860-023-00483-4)
Supplement: Supplementary file 1 — Supplementary Material 1 [file 12860_2023_483_MOESM1_ESM.docx]

**Supplementary file**

**Genetic and protein interaction studies reveal pathway synergy between the ciliary dyslexia candidate genes *DYX1C1* and *DCDC2***

Andrea Bieder, Gayathri Chandrasekar, Arpit Wason, Steffen Erkelenz, Jay Gopalakrishnan, Juha Kere and Isabel Tapia-Páez


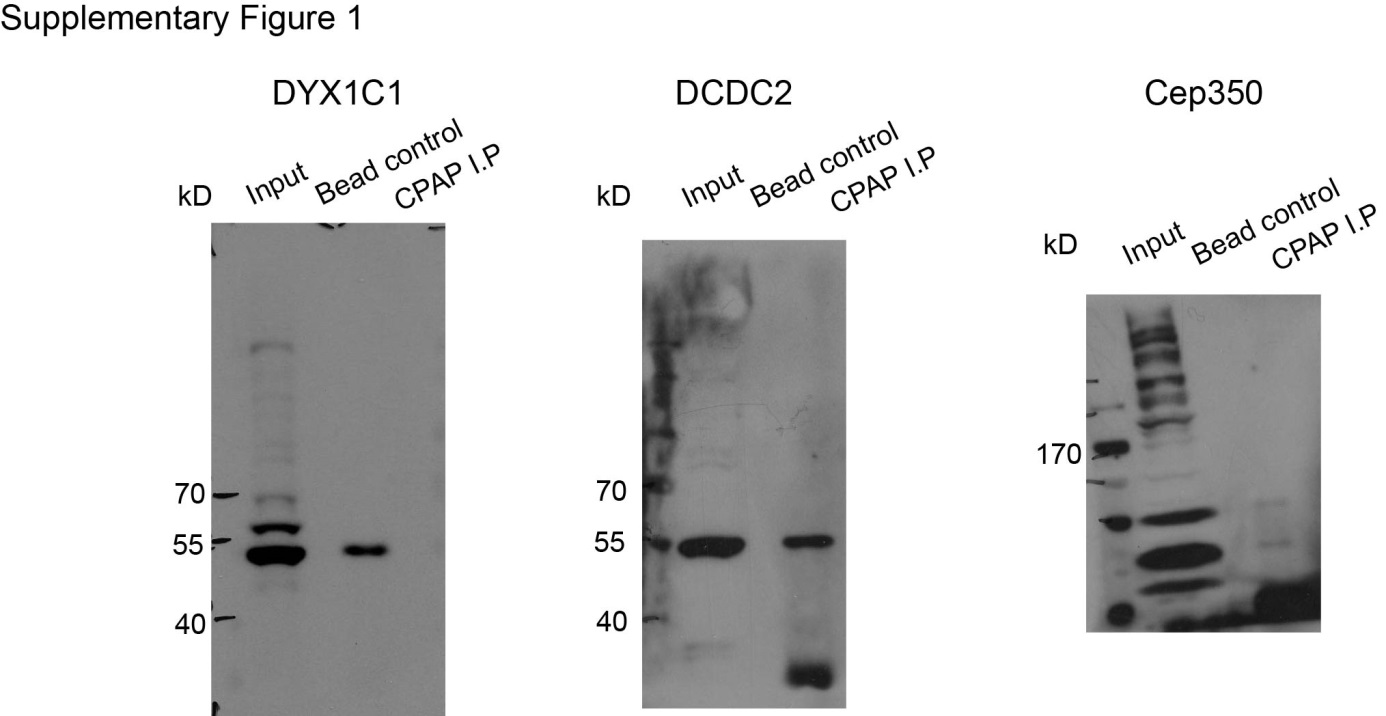


**Supplementary Figure 1:** Full length blots of immunoprecipitations displayed in Figure 1A.

**
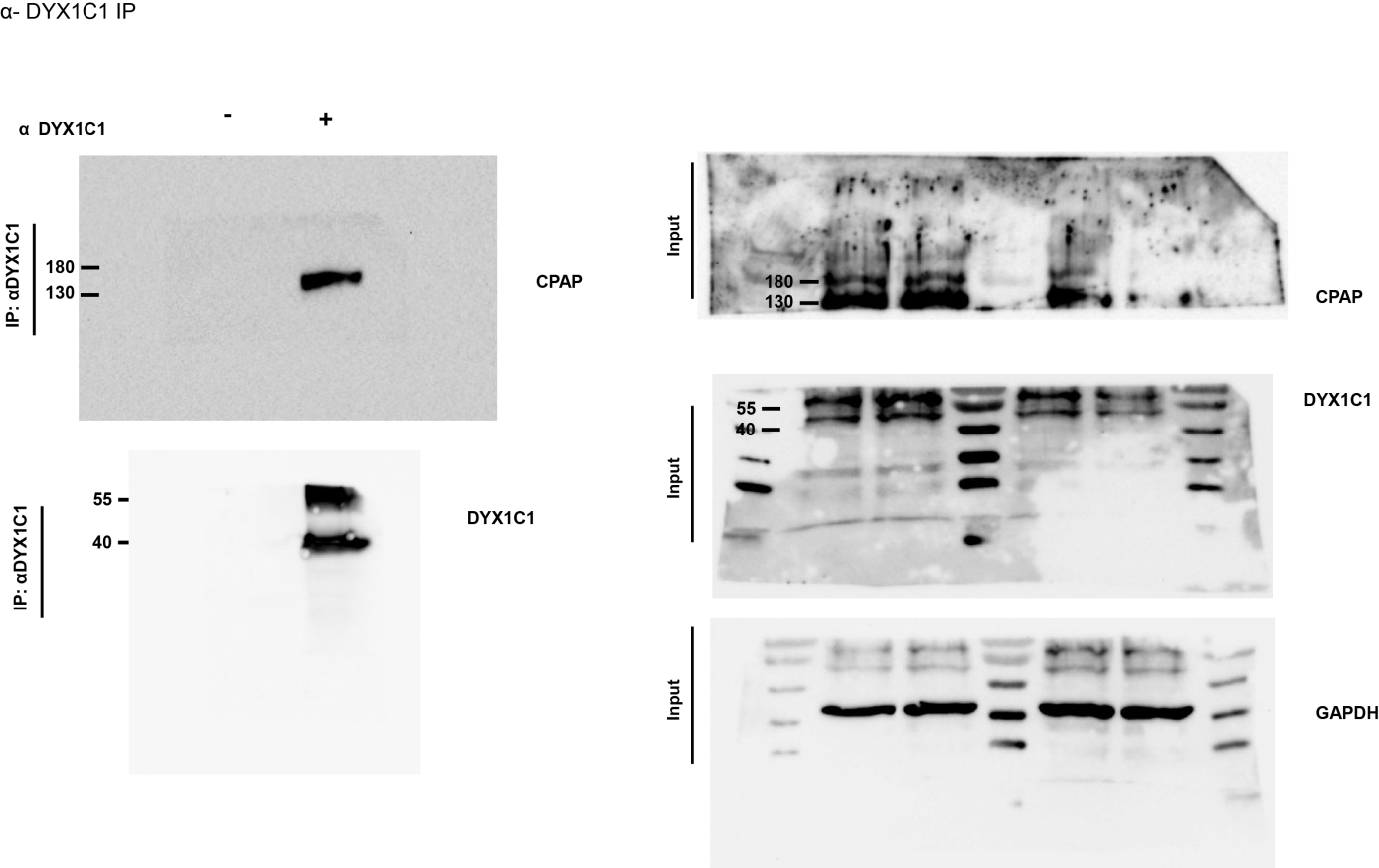
**
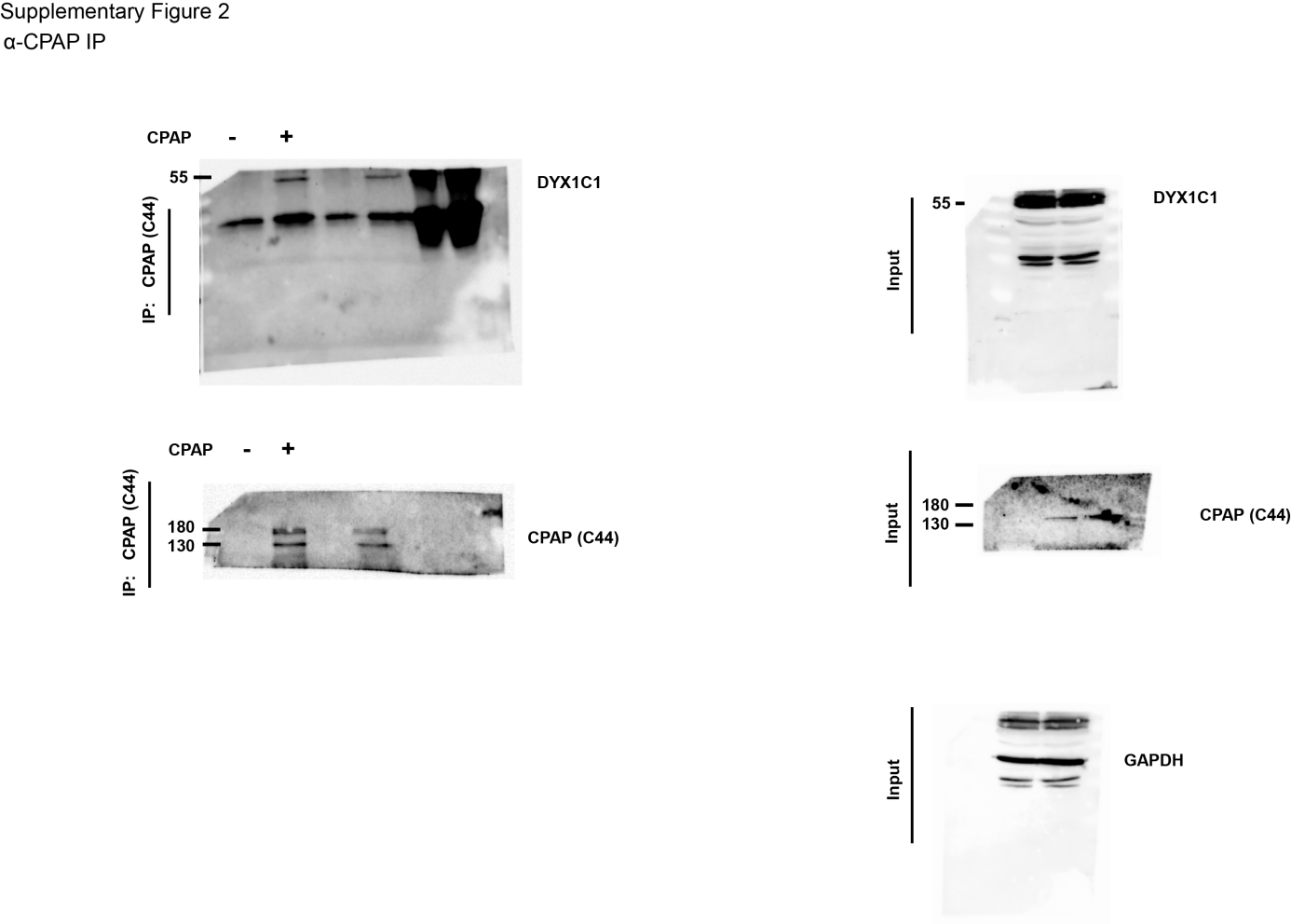


**Supplementary Figure 2:** Full length blots of immunoprecipitations displayed in Figure 1B.
